# Engineering vascularized 3D tissues: An optimized seeding protocol for collagen-based scaffolds

**DOI:** 10.1016/j.mex.2026.103834

**Published:** 2026-02-17

**Authors:** Sophia Walker, Evelyn Hergenhan, Olivia Boerman

**Affiliations:** Biomedical Engineering, Bucknell University, Lewisburg, PA, USA

**Keywords:** 3D culture, Endothelial, Angiogenesis, Cardiovascular, Tissue engineering

## Abstract

The objective of this work was to optimize endothelial cell seeding for 3-dimensional (3D) culture in collagen-based scaffolds to conduct physiologically relevant in vitro studies. Endothelial cells are highly sensitive to their mechanical environment making 2D experiments on polystyrene difficult to translate to in vivo conditions. Therefore, conducting 3D endothelial experiments in collagen scaffolds are critical to elucidate more physiologically accurate responses. However, researchers employ a wide range of scaffold fabrication methods and endothelial cell seeding protocols leading to varying levels of vascularization and interconnectedness between endothelial cells making results difficult to interpret or compare across studies. Here, we propose an optimized endothelial cell seeding protocol using a commercially available collagen scaffold that results in a reproducibly, well-connected vascular network uniformly seeded throughout the entire scaffold enabling researchers to more reliably vascularize collagen scaffolds. We found that pre-saturating scaffolds, seeding from opposing sides of the scaffold, using 600,000 cells per 100 uL of media, and incubating for 120 h after seeding yielded the most uniform and interconnected vascular networks throughout the collagen scaffold.

Our method provides:

An optimized scaffold pre-seeding technique, cell density, and incubation time.

A seeding strategy to ensure consistently vascularized collagen scaffolds with interconnected networks and uniform seeding throughout a commercially available collagen scaffold.

## Graphical abstract

**Figure:**
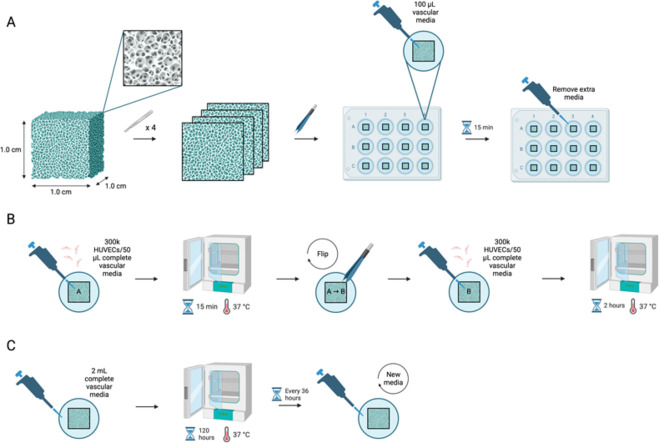

## Specifications table

**Table:** 

**Subject area**	Engineering
**More specific subject area**	In Vitro Mechanobiology and Tissue Engineering Model; Vascular Model on Collagen Scaffold
**Name of your protocol**	Endothelial Cell Seeding Protocol for Three-Dimensional Vascular Network in a Commercial Collagen Scaffold
**Reagents/tools**	Cell line and medium • Primary human umbilical vein endothelial cells, HUVECs (ATCC, Manasses, VA, cat # PCS–100–010) • Vascular cell basal medium (ATCC, cat # PCS–100–030) supplemented with endothelial cell growth kit-VEGF (ATCC, No. PCS–100–041) and 1 % penicillin/streptomycin (ThermoFisher, cat # 15,140,122) Equipment • PHCbi CO_2_ Incubator • Countess 3 Automated Cell Counter (ThermoFisher Scientific) • NuWind Multi-Application Centrifuge • Leica TCS SP5 confocal microscope Reagents • Dulbecco’s phosphate-buffered saline (DPBS, ThermoFisher Scientific, cat # 14,040,182) • Trypsin-EDTA (ATCC, cat # 30–2101) • Cubic collagen-based Spongostan Dental MS0005 (Ethicon, Johnson & Johnson) • 4 % paraformaldehyde (PFA, Boster, cat # AR1068) • Phosphate buffered saline (PBS, ThermoFisher Scientific, cat # 10,010,023) • 1 % bovine serum albumin (BSA, Biophoretics, cat # BCZ11920.1) • Phalloidin (FITC, ThermoFisher Scientific, cat # F432) • 4′,6-diamidino-2-phenylindole (DAPI, Invitrogen, cat # D1306) Software • LAS AF software • ImageJ Angiogenesis Analysis • GraphPad Prism 10.5.0 Plasticware • T75 cm^2^ cell culture flasks (VWR, cat # 10,062–860) • 15 ml conical centrifuge tubes (Oxford Lab Products, cat # OCT-15B) • 0.65 mL microcentrifuge tubes (VWR, cat # 87,003–290) • 12-well plates (VWR, cat # 10,861–556) • Scalpel handle #3 • Sterile surgical carbon steel blades (P&P Medical Surgical, size 10) • Tweezers • 20 μL sterile pipette tips (Oxford Lab Products, cat # XR-20-SLF) • 200 μL sterile pipette tips (Oxford Lab Products, cat # XR-200-SLF) • 1000 μL sterile pipette tips (Oxford Lab Products, cat # XR-1000-SLF) • 20 μL pipette (Eppendorf, cat # ES-20F) • 200 μL pipette (Eppendorf, cat # ES-200F) • 1000 μL pipette (Eppendorf, cat # ES-1000F) • 5 mL serological pipettes (Oxford Lab Products, cat # OSC-5C) • 10 mL serological pipettes (Oxford Lab Products, cat # OSP-10C)
**Experimental design**	Endothelial cells were seeded in a commercial 3D collagen scaffold to achieve a vascular network. Scaffolded cells were dyed with DAPI and FITC to visualize branching, which was further quantified using ImageJ Angiogenesis Analysis. Scaffold saturation, cell density, one-sided and two-sided seeding, and incubation period were altered to determine the optimal method for achieving maximal vascularization and branching.
**Trial registration**	Not applicable
**Ethics**	Not applicable
**Value of the Protocol**	• This study provides a robust and widely accessible seeding protocol for culturing human umbilical vein endothelial cells (HUVECs) to form vascular networks in vitro in a 3-dimensional (3D) collagen scaffold to enable more physiologically relevant experiments. • The development of a standardized 3D endothelial cell culture method is essential for field-wide consistency across angiogenic studies that aim to mimic the natural extracellular matrix environment in soft tissue. • Studies across literature utilizing endothelial cardiovascular constructs vary in parameters, while these results offer a standardized protocol for 3D neovascular development according to refined experimental parameters of scaffold handling, cell density, and incubation period.

## Background

Mechanobiology, a sub-field in tissue engineering, underscores the importance of mechanical environment on cell behavior. A physiologically relevant mechanical environment is critical in studying how cells respond to mechanical stimulus [1] making it essential to consider in tissue engineering experiments to maximize in vivo translation. Mechanobiology is an inherently interdisciplinary field, bringing together a wide range of researchers from clinicians to biologists and engineers. The entry point for tissue engineers to in vitro cell work is often through 2-dimensional cell culture due to simplicity. Biju et al outlined the key differences in cell response between 2D and 3D cultures in a recent review which underscored how cells respond to their extracellular environment, interact with other cell types, and change their morphology differently in a 3D environment when compared to a 2D environment. Despite great advancements in 3D cell culture technologies, the authors conclude that researchers are now focusing on standardizing these 3D techniques to become more organized [2].

It is particularly imperative to consider this 3D environment in studying the behavior and integration of vasculature [3], necessary for engineered tissues to successfully integrate biologically. Vasculogenesis, the origination of blood vessels, involves an aggregate of endothelial cells which turn into tubes followed by larger vasculature [4]. Endothelial cells are especially sensitive to their environment and respond to both mechanical stimulus (e.g. shear-stress) and their environment (e.g. the stiffness of their surrounding extracellular matrix) [5]. These mechanical cues regulate behavior and function which can encourage functional vasculo- and angiogenesis or dysregulated endothelial response hampering healthy vasculature [6] leading to favorable or poor engineered tissue integration [7]. Yi et al in their recent overview describe how the extracellular matrix provides critically complex biochemical and biophysical cues that greatly impact cell response [8] making cell-matrix interactions a continued focus for engineering successful biomaterials for regenerative medicine.

Some studies have explored 3D systems to study vascular networks. These scaffold environments span a number of complex biochemical formulations and fabrication methods including a nanofiber scaffold fabricated using electrospinning consisting of electrically spinning nanofibers into sheets collected on aluminum foil gathered to create a scaffold [9], a porous chitosan-gelatin scaffold fabricated by mixing 2 % chitosan and 2 % gelatin and poured into circular molds and freeze-dried later to be cut into discs to fit into a 6-well plate [10], and a bio-printed GELMA scaffold [11]. Given the specialized knowledge and equipment required, these 3D scaffolds remain inaccessible to researchers approaching tissue engineering from non-chemical or fabrication backgrounds, demonstrating a need for protocol utilizing a commercially available product. Moreover, Rofaani, et al. summarized over 25 different scaffold systems used to study vascular endothelial cells in a mini review [12] underscoring the necessity for a standardized protocol. Researchers have begun to establish protocols in an attempt to standardize collagen fabrication methods including protocols for using decellularized plants [13] and casting collagen gels with embedded cells [14]. While these protocols are helpful in standardizing collagen scaffold fabrication for comparable results across studies, they still rely on researchers fabricating scaffolds themselves which may be inaccessible due to access to resources and possibly result in variable outcomes. Moreover, studies omit optimization of cell seeding parameters like cell density and incubation time.

Therefore, the goal of this study was to establish a standardized protocol to develop a vascular network using a widely accessible, commercially available 3D collagen scaffold. We used Ethicon Spongostan, a sterile, water-insoluble, porcine gelatin sponge chosen for its biological compatibility, extracellular matrix similarity to soft tissue which is largely comprised of collagen, and its stiffness, when saturated in media, more closely mimicking the stiffness of soft tissue than polystyrene. We varied preparation techniques, cell density, and incubation time for the optimal set of parameters. A well-connected vascular network was determined by the number of vascular junctions, vascular length in pixels, and master segment length in pixels. Our methodology ensures uniformly, well-connected vascularized collagen scaffolds across samples while utilizing a commercially available collagen scaffold that is accessible and reproducibly fabricated. In optimizing this protocol, we hope to encourage and increase the number of studies using 3D environments making 3D vascularized cultures more accessible, allow for more direct comparison across publications, increase the quality and quantity of physiologically relevant data in mechanobiology, and ultimately increase the potential success of tissue engineering therapeutics in vivo.

## Description of protocol

Unless otherwise noted, all steps were performed under sterile conditions in a biosafety cabinet in a BSL 2 facility.

### Step 1: cell culture

Primary human umbilical vein endothelial cells, HUVECs, were cultured in vascular media between passages 2 and 9 in 37 °C and 5 % CO_2_ until 70–80 % confluency before experimental use.

5 mL and 10 mL serological pipettes were used to add and remove liquid in all Step 1 procedures.

1. Media was removed from flasks.
2. Cells were washed using 5 mL DPBS.
3. Cells were detached using 3 mL trypsin, incubating for 5 min.
4. Cells were quenched in 7 mL complete vascular media.
5. 10 μL of the cell suspension was mixed with 10 μL of trypan blue (1:1) in a 0.65 mL microcentrifuge tube using a 20 μL pipette and counted using the cell counter (in non-sterile conditions).
6. The cell suspension was centrifuged at 125 g for 6 min at room temperature to form a cell pellet (Fig. 1A).

**Fig. 1:**
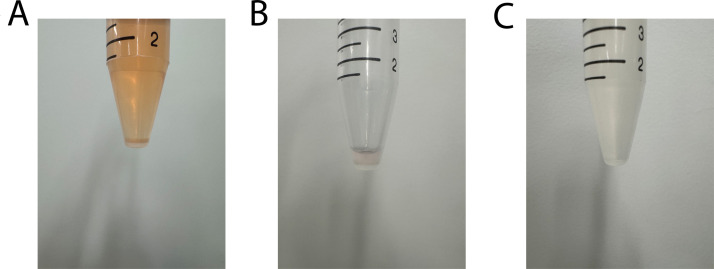
(A) Cell pellet following centrifuging lifted cells. (B) Cell pellet remaining after supernatant was aspirated and discarded. (C) Cell pellet resuspended in vascular media (volume of vascular media used will vary depending on cell count and desired cell seeding concentration).
7. The supernatant containing trypsin was carefully aspirated and discarded (Fig. 1B).
8. The cell pellet was resuspended in a calculated volume of complete vascular media for the desired cell seeding concentration (Fig. 1C).

### Step 2: 3D collagen scaffold preparation

The Spongostan scaffold, an absorbable hemostatic gelatin sponge made from sterile porcine gelatin (1.0 cm^3^), was chosen as the scaffold of interest for its extracellular composition and stiffness more closely mimicking that of soft tissue as compared to polystyrene. This scaffold contains micropores of 60.66 ± 24.48 μm diameter and nanopores of 32.97 ± 1.41 nm [15], permitting appropriate retention of HUVECs according to their approximate 17 μm diameter [16]. Moreover, this commercially sold scaffold is widely available demonstrating less sample-to-sample variability as compared to individual lab-based formulations.

1. Scaffolds were evenly cut into four 25 μm thin slices (Fig. 2A and 2B) using a scalpel to adhere to the confocal lens working distance, which may differ depending on the available confocal microscope. Scaffolds can be cut on the inside surface of the lid to a sterile 12-well plate.

**Fig. 2:**
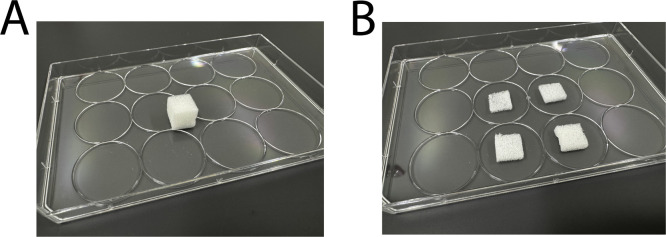
Scaffolds (A) prior to and (B) after being cut into quarter slices.
2. Scaffold slices were transferred using sterilized tweezers into their own untreated well in a sterile 12-well plate (Fig. 3A).

**Fig. 3:**
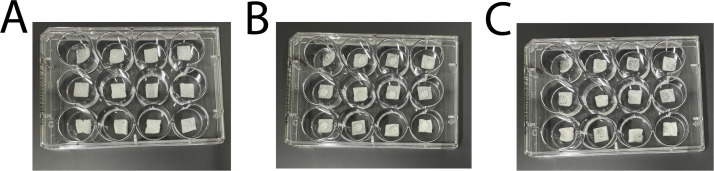
Scaffolds (A) placed into a sterile 12-well plate, (B) at the beginning of pre-saturation and (C) after pre-saturation and excess media has been aspirated.
3. Scaffold slices intended for pre-saturation were pre-saturated using 100 μL of complete vascular media, measured using a 200 μL pipette, for 15 min at room temperature. Simply pipette the media onto the top of the scaffold in a bead (Fig. 3B). If the bead rolls off or does not fully absorb into the scaffold after the 15 min, move the scaffold around into the media to ensure it soaks up the media.
4. All excess media was aspirated from the well before cell seeding (Fig. 3C).

**Fig. 4:**
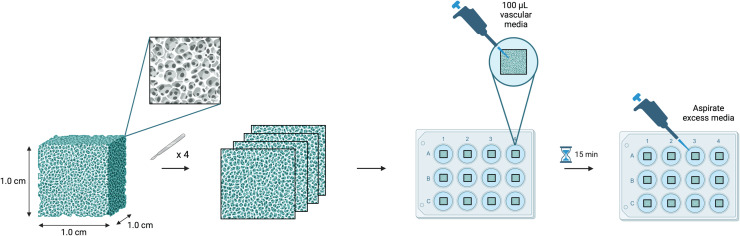
Schematic of recommended preparation for seeding experiments depicting the original scaffold from the manufacturer, slicing into 4 equal sections, placing into 12-well plate with 100 μL of media for 15 min of pre-soaking, and aspiration of excess media.

Fig. 4 shows the collagen scaffold preparation.

To determine if the slicing method produced uniform quarters, three measurements were taken: 1) the thickness of each slice using sterilized calipers, 2) the weight of each slice (dry) using an analytical mass balance, and 3) the weight of each slice after pre-saturation. Three scaffolds were used for a total of twelve slices. Two slices were visually significantly thicker than the others and would have been omitted but were kept in the study for comparison.

### Step 3: cell seeding

1. 50 μL of the desired cell suspension (4 × 10^5^, 6 × 10^5^, and 8 × 10^5^ cells per 100 μL of vascular media) was pipetted directly onto the pre-saturated scaffolds and incubated at 37 °C and 5 % CO_2_ for 2 h.
2. Scaffolds analyzed for 2-sided seeding were flipped from face up to face down using sterilized tweezers.
3. An additional 50 μL of the calculated cell suspension was pipetted directly onto the scaffolds and incubated at 37 °C and 5 % CO_2_ for 2 h for a total of 100 μL of cell suspension seeded onto each scaffold.
4. 2 mL of complete vascular media, measured using a 1000 μL pipette, were added to each scaffold-containing well and incubated for given duration (96, 120, 144, or 168 h (*n* = 24)), replacing the vascular media every 36 h.

**Fig. 5:**
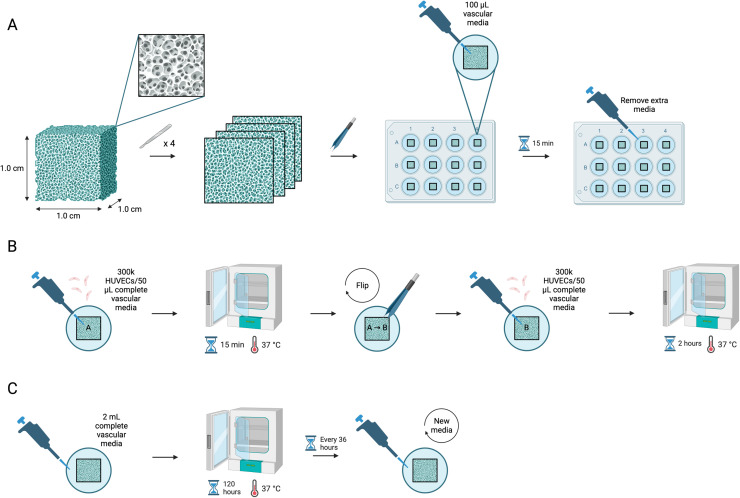
Schematic of recommended scaffold preparation and full seeding protocol depicting the (A) original scaffold, scaffold slicing, pre-soaking for 15 min, aspiration of excess media, (B) seeding cells on side A of the scaffold, incubation for 15 min, then seeding cells on side B of scaffold, incubation for 2 h, (C) adding 2 mLs of media to the well, and 120 h of incubation with media changes every 36 h.

Fig. 5 shows the entire cell seeding methods including scaffold preparation.

### Step 4: immunofluorescence staining and imaging

A 1000 μL pipette was used to pipette liquids in all procedures in Step 4.

1. At the end of each experimental time point, the complete vascular media was aspirated.
2. 1000 μL of 4 % PFA was added to each cell-seeded scaffold for 15 min at room temperature to fix the cells.

All following steps can be performed in non-sterile conditions at room temperature, covered from light by wrapping the plate in tin foil and dimming all lights to the lowest setting.

1. Scaffolds were rinsed three times with 2 mL PBS.
2. Scaffolds were then blocked for 60 min with 1000 μL of 1 % BSA reconstituted in PBS.
3. Scaffolds were rinsed three times with 2 mL PBS.
4. Scaffolds were stained for nuclei using 2 mL DAPI (1:30,000 in PBS) for 30 min.
5. Scaffolds were rinsed three times with 2 mL PBS.
6. Scaffolds were stained for F-actin using 2 mL FITC (1:5000 in PBS) for 1 hour.
7. Scaffolds were rinsed three times with 2 mL PBS.
8. Scaffolds were stored in 2 mL PBS at 4 °C until imaging.
9. Scaffolds were imaged within 36 h of staining using the confocal microscope with LAS AF software to acquire images at 40X. A minimum of 3 images were captured for each scaffold.

### Step 5: imageJ and statistical analysis

Images were analyzed using the ImageJ Angiogenesis Analysis software, specifically for fluorescence images, to quantify angiogenic metrics such as vascular junction quantity (bifurcation of multiple nodes), total vascular length (the sum of the lengths of all network segments), and master segment length (the sum of the lengths of network segments connecting at least two junctions that each link at least three segments) for each incubation period. A one-way ANOVA was used to determine statistical significance followed by Tukey’s Post Hoc analysis using GraphPad Prism. A p-value of less than 0.05 was considered to be significant.

## Protocol validation

### Scaffold uniformity

**Fig. 6:**
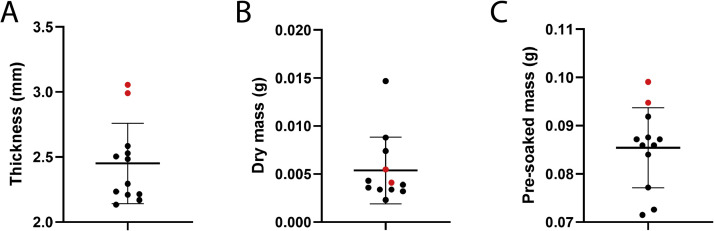
(A) Thickness, (B) dry mass, and (C) mass after pre-saturation with 100 μL of vascular media of quarter-sliced scaffolds. *n* = 12 with 2 scaffolds that would have been discarded for being too thick after visual inspection (red). The bars show the mean and standard deviation of the data.

Scaffolds were manually sliced into four even sections and 12 scaffold slices were collected to test for uniformity across slices. Each scaffold slice was measured for its thickness using calipers and mass using an analytical scale. Our results demonstrate a slight variance between slices; however, scaffolds are largely uniform. There were two visually thicker slices, indicated in red in Fig. 6, that is recommended to be omitted from use in experiments. The ten other scaffolds measured within one standard deviation of the mean, with the thicker, omitted scaffolds falling outside of this range. Overall, the two thicker, exclusionary slices are clearly much thicker and heavier after soaking. The dry mass of these exclusionary, thicker slices is within the standard deviation of other slices; however, this is likely due to standard error of the analytical scale when measuring such small masses. We recommend researchers to omit visually thicker slices that may occur on occasion depending on training level and familiarity with the technique of slicing. The marginal differences in scaffold thickness for the remaining scaffolds do not make a significant impact on overall thickness or results. Therefore, the method of slicing into four even sections using a scalpel allows for relatively uniform slices (Fig. 6).

### 3D Collagen scaffold preparation

**Fig. 7:**
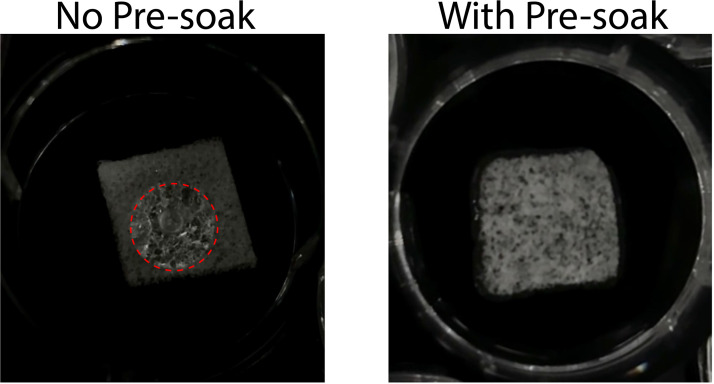
Representative images of a scaffold without (left) and with pre-soaking (right). Without pre-soaking, the media beaded on top of the scaffold, demarcated with the dashed red circle, and did not permeate through the scaffold. With pre-soaking, the media uniformly distributed throughout the scaffold.

Sliced scaffolds were then either pre-soaked with 100 uL of complete vascular media for 15 min or seeded with cells without this pre-soak making the scaffold entirely dry during seeding. Scaffolds that were not pre-saturated did not fully absorb the seeded cell suspension leading to uneven seeding and poor vascular network throughout the scaffold. Scaffolds that were pre-saturated with 100 μL of complete vascular media for 15 min did fully and evenly absorb the cell suspension, leading to even vascular networking (Fig. 7).

### Cell seeding: one-sided vs. two-sided seeding

**Fig. 8:**
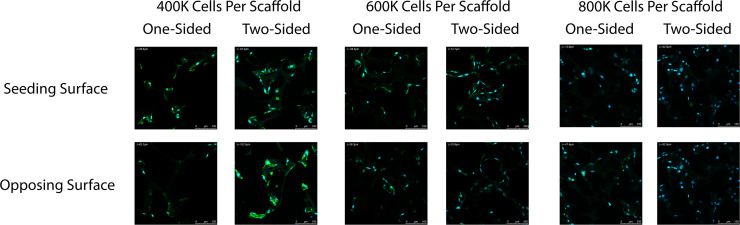
Representative confocal images of the seeding surface and opposing surface of scaffolds seeded with either 100 μL of cell suspension on one-side or 50 μL of cell suspension on both top and bottom (opposing sides) of the scaffold. 400,000, 600,000, and 800,000 total cells per scaffold were tested. Cells were stained for their nuclei (blue) and cytoskeleton actin (green).

**Fig. 9:**
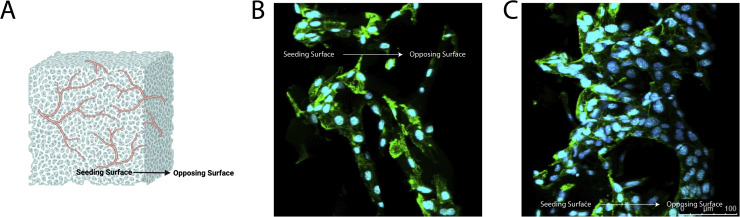
Representative (A) angled confocal images of a z-stack projection of scaffolds seeded with either (B) 100 μL of cell suspension on one-side or (C) 50 μL of cell suspension on both top and bottom (opposing sides) of the scaffold. Cells were stained for their nuclei (blue) and cytoskeleton actin (green).

Scaffolds were then seeded either from one side or two sides. Scaffolds seeded with 100 μL of cell suspension on one side resulted in partial cell migration through the scaffold and yielded a visibly denser network on one side and sparse networking on the opposing scaffold side. In contrast, scaffolds seeded with 50 μL of cell suspension on one side, incubated for 2 h, then seeded with another 50 μL of cell suspension on the opposing side resulted in a more evenly distributed vascular network longitudinally throughout the scaffold (Fig. 8, Fig. 9).

### Cell density and incubation time

**Fig. 10:**
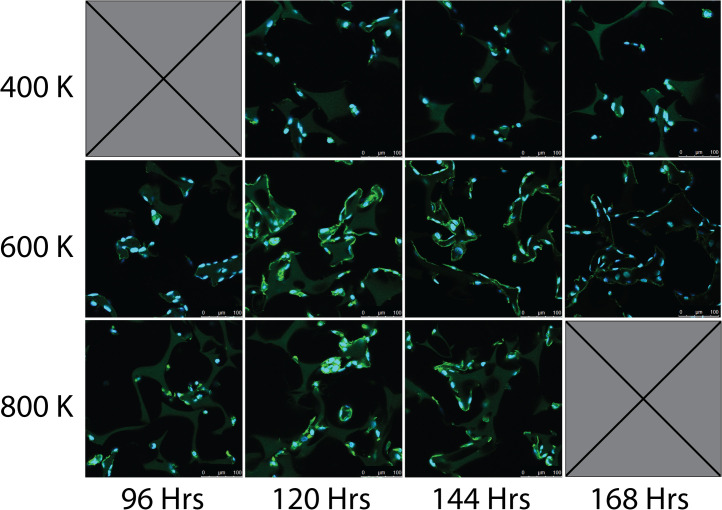
Representative confocal images of cell seeded scaffolds at 400,000, 600,000 or 800,000 cells per scaffold and 96, 120, 144, or 168 h after cell seeding. Cells were stained for their nuclei (blue) and cytoskeleton actin (green).

**Fig. 11:**
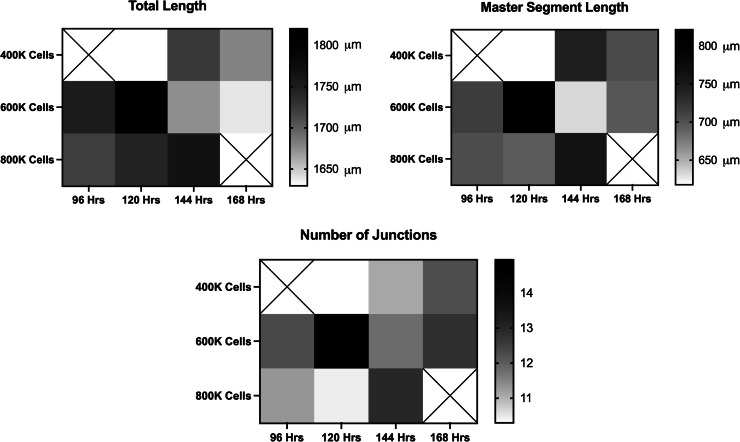
Average total vascular length, master segment length, and number of junctions of vascular networks at 96, 120, 144, and 168 h of incubation formed by 400,000, 600,000, and 800,000 cells per scaffold by heatmap. *n* = 24..

Cell density and incubation times were then assessed for the most optimal cell seeding parameters for interconnected vascular networks. Evaluated parameters included cell densities of 400,000, 600,000, and 800,000 cells per 100 µL, and incubation periods of 96, 120, 144, and 168 h. As seen in the representative raw images at each cell density in Fig. 10, 4 × 10^5^ cells qualitatively resulted in a vascular network that was sparse and resulted in fully connected vasculature at all time points. Seeding a cell density of 8 × 10^5^ cells per scaffold resulted in a vascular network that was too dense to form distinguishable vessels or networks also at all time points. Contrastingly, a cell density of 6 × 10^5^ cells per scaffold resulted in an evenly distributed established vascular network throughout the scaffold. This is supported quantitatively in Fig. 11 where the total vascular length, master segment length, and number of master junctions are highest in the 6 × 10^5^ cells per scaffold group as compared to 4 × 10^5^ and 8 × 10^5^ cells per scaffold groups. Moreover, the total vascular length, master segment length, and number of master junctions are highest 120 h after cell seeding when compared to 96, 144, and 168 h making 120 h the most optimal incubation time for vascularization.

## Limitations

Although our study results in consistently well developed and interconnected vascular networks, future studies should include a more comprehensive study of material properties to understand how they affect vascularization. Some of these parameters may include scaffold degradation rate and stiffness which have shown to impact cell vascularization [17]. Moreover, future iterations of this seeding protocol could include co-cultures which would make this an even more physiologically relevant model. For soft tissue, some of those key cells may include fibroblasts, macrophages, or keratinocytes. Due to the relatively high pore size in the scaffold, the seeding protocol of these smaller-sized cells will need to be further optimized. Lastly, future studies could employ more robust quantitative characteristics evaluating the vascular networks using more sophisticated image analysis tools including but not limited to volume-based metrics as described in Shirazi et al. (2019) [18] These considerations notwithstanding, this study provides a robust and widely accessible protocol for researchers to conduct 3D endothelial studies enabling more physiologically relevant experiments in the field.

## Related research article

None

## CRediT authorship contribution statement

**Sophia Walker:** Methodology, Software, Validation, Formal analysis, Investigation, Data curation, Writing – original draft, Visualization. **Evelyn Hergenhan:** Validation, Formal analysis, Investigation, Writing – original draft. **Olivia Boerman:** Conceptualization, Methodology, Resources, Data curation, Writing – review & editing, Visualization, Supervision, Project administration, Funding acquisition.

## Declaration of competing interest

The authors declare that they have no known competing financial interests or personal relationships that could have appeared to influence the work reported in this paper.

## Data Availability

No data was used for the research described in the article.
